# Type III CRISPR-based RNA editing for programmable control of SARS-CoV-2 and human coronaviruses

**DOI:** 10.1093/nar/gkac016

**Published:** 2022-02-15

**Authors:** Ping Lin, Guanwang Shen, Kai Guo, Shugang Qin, Qinqin Pu, Zhihan Wang, Pan Gao, Zhenwei Xia, Nadeem Khan, Jianxin Jiang, Qingyou Xia, Min Wu

**Affiliations:** Biological Science Research Center, Southwest University, Chongqing 400715, China; Wound Trauma Medical Center, State Key Laboratory of Trauma, Burns and Combined Injury, Daping Hospital, Army Medical University, Chongqing 400042, China; Biological Science Research Center, Southwest University, Chongqing 400715, China; State Key Laboratory of Silkworm Genome Biology, Southwest University, Chongqing 400716, China; Department of Neurology, University of Michigan, Ann Arbor, MI 48109, USA; Department of Biomedical Sciences, School of Medicine and Health Sciences, University of North Dakota, Grand Forks, ND 58203, USA; Department of Biomedical Sciences, School of Medicine and Health Sciences, University of North Dakota, Grand Forks, ND 58203, USA; Department of Biomedical Sciences, School of Medicine and Health Sciences, University of North Dakota, Grand Forks, ND 58203, USA; Department of Biomedical Sciences, School of Medicine and Health Sciences, University of North Dakota, Grand Forks, ND 58203, USA; Department of Pediatrics, Ruijin Hospital affiliated to Shanghai Jiao Tong University School of Medicine, Shanghai, China; Department of Biomedical Sciences, School of Medicine and Health Sciences, University of North Dakota, Grand Forks, ND 58203, USA; Wound Trauma Medical Center, State Key Laboratory of Trauma, Burns and Combined Injury, Daping Hospital, Army Medical University, Chongqing 400042, China; Biological Science Research Center, Southwest University, Chongqing 400715, China; State Key Laboratory of Silkworm Genome Biology, Southwest University, Chongqing 400716, China; Department of Biomedical Sciences, School of Medicine and Health Sciences, University of North Dakota, Grand Forks, ND 58203, USA

## Abstract

Gene-editing technologies, including the widespread usage of CRISPR endonucleases, have the potential for clinical treatments of various human diseases. Due to the rapid mutations of SARS-CoV-2, specific and effective prevention and treatment by CRISPR toolkits for coronavirus disease 2019 (COVID-19) are urgently needed to control the current pandemic spread. Here, we designed Type III CRISPR endonuclease antivirals for coronaviruses (TEAR-CoV) as a therapeutic to combat SARS-CoV-2 infection. We provided a proof of principle demonstration that TEAR-CoV-based RNA engineering approach leads to RNA-guided transcript degradation both *in vitro* and in eukaryotic cells, which could be used to broadly target RNA viruses. We report that TEAR-CoV not only cleaves SARS-CoV-2 genome and mRNA transcripts, but also degrades live influenza A virus (IAV), impeding viral replication in cells and in mice. Moreover, bioinformatics screening of gRNAs along RNA sequences reveals that a group of five gRNAs (hCoV-gRNAs) could potentially target 99.98% of human coronaviruses. TEAR-CoV also exerted specific targeting and cleavage of common human coronaviruses. The fast design and broad targeting of TEAR-CoV may represent a versatile antiviral approach for SARS-CoV-2 or potentially other emerging human coronaviruses.

## INTRODUCTION

COVID-19, caused by severe acute respiratory syndrome-coronavirus-2 (SARS-CoV-2), has led to an unprecedented global health crisis (1–3). No specific antivirals for SARS-CoV-2 are currently available for patients in the clinics (4). Moreover, recent reports demonstrate that SARS-CoV-2 is evolving with divergent mutations presenting daunting challenges for the design of antivirals and vaccines (5). Rising technologies, such as CRISPR-Cas systems, have emerged as potential antiviral strategies by targeting viral genome and transcripts, resulting in degradation of the genome and gene products, which may repress virus replication and spread (6,7).

Both type III and VI CRISPR-Cas system mediate RNA-guided RNA cleavage. RNA-guided RNA cleavage by Cas13 was recently used for detection and inhibition of RNA viruses (6,7). However, activated Type VI systems in cells would cleave both crRNA-bound target RNA (*cis*-cleavage) and non-target RNAs (trans-cleavage) (8,9), inducing collateral activity (non-specific targeting host transcriptome, also called off-target effects) and potential cytotoxicity (10). This limitation hampers Cas13 therapeutic development for clinical application for COVID-19 patients. In addition, other forms of therapeutics for COVID-19 are highly needed due to continued viral mutations and very few effective specific treatments are currently in place.

**Figure 1.**
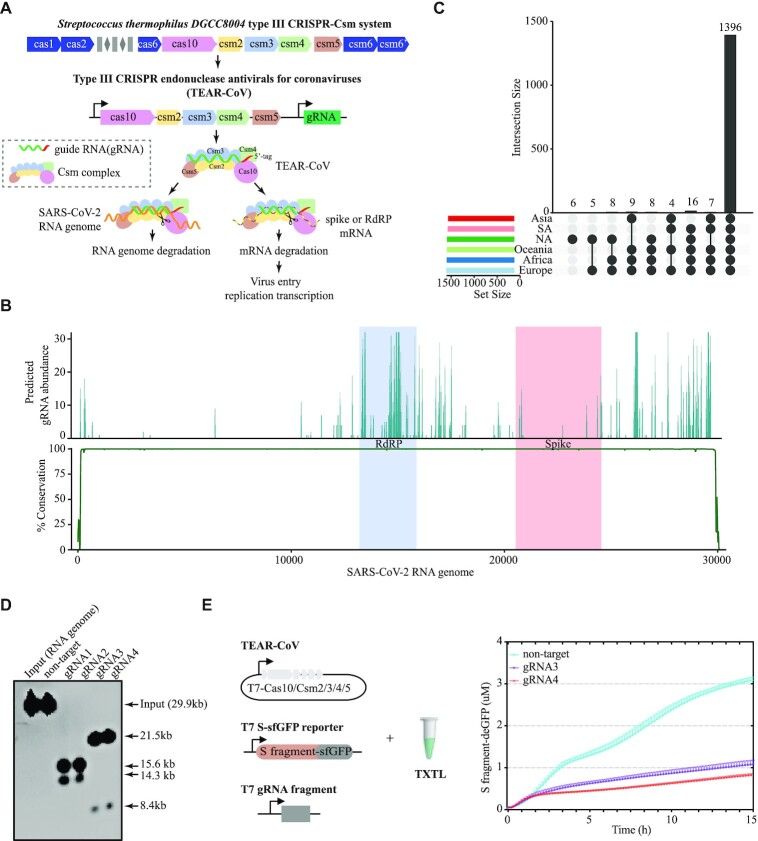
TEAR-CoV approach inhibits SARS-CoV-2 using a simplified type III CRISPR-Csm system. (**A**) Type III CRISPR endonuclease antivirals for coronaviruses (TEAR-CoV) derived from *Streptococcus thermophilus* type III CRISPR-Cas system to limit SARS-CoV-2 replication and infection by directly targeting and degrading the viral RNA genome and reduction of viral mRNAs. (**B**) Conserved regions of SARS-CoV-2 sequences with SARS-CoV and MERS-CoV. Top: gRNA abundance in the highly conserved regions that target SARS-CoV-2 sequences and SARS or MERS. Bottom: percentage of conservation between aligned viral genomes. (**C**) A collection of gRNAs targeting SARS-CoV-2 strains derived from Asia, South America (SA), North America (NA), Oceania, Africa, and Europe. The Y axis represents the number of gRNAs targeting SARS-CoV-2. Upset plot of each area overlaps across the six continents. (**D**) Cleavage assay of the SARS-CoV-2 RNA genome by TEAR-CoV *in vitro*. gRNA1 and gRNA2 target the RdRP sequence; gRNA3 and gRNA4 target the S sequence; and not-target represents a gRNA not targeting the SARS-CoV-2 RNA genome. (**E**) Transcription-translation (TXTL) reaction assessing TEAR-CoV in repression of S fragment-deGFP expression. Left lane: overview of the TXTL schematic; plasmid Csm complex (Cas10-Csm2-Csm3-Csm4-Csm5), S fragment fused GFP fluorescent reporter, and a T7-gRNA fragment. Right lane: kinetic GFP fluorescence measured over the course. Bars indicate mean SEM.

Type III CRISPR-Cas system can recognize and cleave both RNA and DNA. Type III-A system is composed of Cas1, Cas2, Cas6, Cas10 and Csm2/3/4/5/6/6′ (Figure 1A) (11,12). The Csm complex (composed of Cas10, Csm2, Csm3, Csm4 and Csm5 proteins) together with CRISPR RNAs (crRNAs) assemble into Csm-crRNA complex to degrade specific-target RNA (11). Binding to a target RNA increases the conformational flexibility of Cas10, activating its ssDNA cleavage activity (13,14). On contrast, an ‘anti-tag’ RNA, containing both a matching protospacer sequence and a 3′-flanking sequence complementary to crRNA 5′-tag, prevents Cas10 from formulation of the DNase-active state, and thereby inhibiting the non-specific ssDNA degradation and avoiding autoimmunity (13,15,16). Csm6 RNase is activated for non-specific cleavage of RNAs upon the production of cyclic oligoadenylates (cOAs) that are synthesized by the activated Cas10 (17). This nonspecific RNA targeting can be toxic to cells (Supplementary Figure S1) (12,13,18), which may inflict off-target effects and potentially causing host cell transcriptome degradation (Supplementary Figure S1) (10,19). Hence, native type III CRISPR are not tested for editing human genes or devising prophylactic or therapeutic options for diseases.

Here, we attempted to harness the unique functionality of Type III CRISPR to design an antiviral biotherapy, Type III CRISPR endonuclease antivirals for coronaviruses (TEAR-CoV) to combat the pandemic-causing RNA virus SARS-CoV-2, influenza A virus (IAV) and human coronaviruses. We first explored the broad utility of TEAR-CoV for targeting and degrading the SARS-CoV-2 sequence by computational analysis. We then experimentally tested TEAR-CoV’s ability to cleave SARS-CoV-2 genome and mRNA transcripts and degrade live IAV to block viral replication. Finally, we explored that a group of gRNAs combined with TEAR-CoV may sufficiently degrade almost all human coronaviruses. Our data suggest that this approach may exert the precise antiviral function to an emerging virus in a timely manner, which may be useful for the preparedness for future pandemics.

## MATERIALS AND METHODS

### Download genome sequence

All SARS-CoV-2 genome sequences were downloaded from https://www.ncbi.nlm.nih.gov/sars-cov-2/ on 26 July 2020. After removing very short genome and the genome containing gap and potential unknown sequences, the complete SARS-CoV-2 reference genomes were used for bioinformatic analysis. All human coronavirus complete genome sequences were downloaded from Virus Pathogen Resource database on 30 July 2020.

### Predicted conserved gRNA sequences for SARS-CoV-2

To screen possible gRNAs for targeting SAR-CoV-2 virus, as well as SARS-CoV and MERS-CoV, the highly conserved regions were aligned by MAFFT using the-auto flag. A 32-nucleotide (nt) fragment as possible gRNAs was extracted from the highly conserved regions with coverage larger than 0.5 among SARS-CoV-2 genomes to collect enough potential gRNAs with a sliding window approach, as well as with 1 mismatch to SARS-CoV or MERS-CoV reference genomes. Next, we performed the alignment of gRNAs to human transcriptome to avoid gRNA targeting any sequences in the human genome (HG 38, including non-coding RNA). Studies suggest that mismatch combinations are often tolerated in the overall genome's transcripts, with <5 mutations (<5 mismatches) at the position +1 to +5 in 32nt gRNAs significantly allowing RNA binding and degradation by Type III system (15,16). We propose to avoid using gRNAs with <2 nt preferably three or four mismatches in the position +1 to +5 through the bowtie to minimize the possibility of degrading human transcripts (15,16,20).

### Bioinformatic analysis of gRNA pool targeting human coronaviruses

Considering preparedness for future pandemics, we attempted to generate a minimal number of hCoV-gRNAs’ combination that may be sufficient to target all known human coronaviruses (species and strains) based on the strategy described by Abbott *et al.* (6). The computational pipeline was adjusted to extract all unique 32nt sliding windows in human coronaviruses’ genomes, yielding gRNA candidates. The minimal number required for effectively targeting all human coronaviruses’ genomes were generated by mapping each gRNA candidate with a perfect match. The hCoV-gRNA1 targeting the most genomes was added to minimal gRNA pool. After removing the hCoV-gRNA1 targeting genome, hCoV-gRNA2 was extracted from remaining human coronavirus with most genome targeting and added to minimal gRNA pool. We repeated this iteration until a set of hCoV-gRNAs targeting almost all human coronavirus genomes was attained. The phylogenetic tree and its-associated annotation were generated by Interactive Tree of Life (iTOL v5.5; https://itol.embl.de/).

### Microbes

*Escherichia coli* DH5α, BL21 and its-derived strains were routinely grown in LB or LB-agar media and, when required, supplemented with appropriate antibiotic(s), such as kanamycin and streptomycin.

### Protein expression and purification

The pCas10/Csm plasmid was provided by Dr Zhiwei Huang from Harbin Institute of Technology and was used to make each subunit of Csm complexes into pET28a (pET28a-Cas10, pET28a-Csm2, pET28a-Csm3, pET28a-Csm4 and pET28a-Csm5) with kanamycin resistance. Each recombinant plasmid was transfected into *E. coli* BL21 (New England Biolabs, Ipswich, MA). *E. coli* BL21 with pET28a-Cas10 strain, *E. coli* BL21 with pET28a-Csm2 strain, *E. coli* BL21 with pET28a-Csm3 strain, *E. coli* BL21 with pET28a-Csm4 strain, and *E. coli* BL21 with pET28a-Csm5 strain were cultured at 28°C, induced by IPTG to express each subunit of Csm complexes, and purified by using Ni^2+^-NTA resin. Briefly, *E. coli* BL21 containing the recombinant plasmid was cultured in LB media with OD_600_ = 0.5 and then induced with 0.5 mM IPTG for 8 h at 28°C. Cells were lysed by sonication in a lysis buffer (Phosphate Buffered Saline, pH 7.2, two complete EDTA-free protease inhibitor tablets, 1 mM phenylmethyl-sulfonyl fluoride). Cell debris was removed by centrifugation at 15 000 g/min for 10 min at 4°C. His-tagged proteins were purified using Ni^2+^-NTA resin (Thermo Fisher Scientific, Rockford, IL) according to the manufacturer's instructions. After dialysis, the proteins were concentrated through Pierce^TM^ Protein Concentrator PES-3K MWCO (Thermo Fisher Scientific, Waltham, MA) and detected by SDS-PAGE gels.

### Cleavage activity assays

Mature 40-nt gRNAs for targeting the RdRP or S regions were synthesized from Integrated DNA Technologies (IDT, Coralville, IA). SARS-CoV-2 RNA genome was obtained from ATCC. *In vitro* RNA cleavage reaction: The StCsm complexes (Cas10, Csm2, Csm3, Csm4 and Csm5) were mixed with 40-nt gRNA (1:1, 500 nM) in a cleavage buffer [25 mM Tris–HCl (pH 8.0), 2 mM MgCl_2_, 60 mM NaCl] and incubated for 15 min. Following complex formation, the SARS-CoV-2 RNA genome was added to the StCsm-gRNA effector complex at 4 μM. The reactions were incubated at 37°C for 60 min, run on 0.8% agarose gels (1× running MOPS buffer and formaldehyde), and visualized by EB staining.

### The transcription–translation (TXTL) reactions *in vitro*

The TXTL reaction system used in this study is myTXTL T7 expression kit from Arbor Biosciences (Ann Arbor, MI). DNA plasmids or fragments used for TXTL reaction included, T7-mini type III system plasmid (T7-Cas10/Csm2/3/4/5), an S protein CTD-gRNA amplicon (T7 gRNA fragment), and an S CTD-sfGFP amplicon (T7 S-sfGFP reporter). To prepare plasmids for TXTL reaction, the plasmids were isolated using the QIAGEN Plasmid Mini Kit (QIAGEN, Redwood City, CA) to elute DNA plasmid with 200 μl nuclease-free H_2_O. Using the 200 μl of AMPure XP beads (Beckman Coulter, Brea, CA), we purified the plasmids and fragments according to the handbook by adding 20 μl of nuclease-free H_2_O to elute the plasmids.

The reactions were set up in a total of 12 μl following the manufacturer's instructions. Each reaction contained 9 μl of TXTL master mix, 0.125 nM of T7 S fragment-deGFP reporter, 1 nM of T7-Cas10/Csm2/3/4/5, 2 nM of T7 gRNA fragment and 2.5 μM of β-d-1-thiogalactopyranoside (IPTG). The reactions were carried out to measure GFP fluorescence value through BioTeK Synergy HT Multi-Mode Microplate Reader with excitation 485 nm, emission 528 nm at 29°C for up to 15 h. The GFP concentration was calculated according to the manufacturer's instructions through the eGFP standard curve. In brief, we prepared a 2-fold dilution series of eGFP (Cell Biolabs) in the concentration range of 0–5 μM in 1.5 ml tubes. For each dilution, we transferred 10 μl to a 96-well plate to perform eGFP fluorescence measurement using a BioTeK Synergy HT Multi-Mode Microplate Reader. The fluorescence values of each standard protein were used to create an eGFP standard curve. The Y-axis is the fluorescence values of the eGFP standard against their respective protein concentration (X-axis) and fitting the curve to the linear regression formula (*Y* = 2109.5*X*; *R*^2^ = 0.98) to determine the GFP concentration.

### Cell lines

Human Embryonic Kidney 293 plus T cell antigen (HEK293T cells, CRL-3216, ATCC) cells, Vero E6 cells and A549 cells were cultured in RMPI 1640 (Thermo Fisher Scientific) and DMEM (Thermo Fisher Scientific) supplemented with 10% fetal bovine serum (VWR) and penicillin–streptomycin (Thermo Fisher Scientific).

### Lentiviral packaging

To produce SARS-CoV-2-RdRP-GFP lentivirus, HEK293T cells were transfected by SARS-CoV-2-RdRP-GFP (provided by Dr Lei S. Qi), psPAX2 (Addgene #12260) and pMD2.G (Addgene #12259) plasmids. On day 1, HEK293T cells were seeded into 10 cm tissue culture plates in antibiotic-free DMEM media with 10% FBS. On day 2, the cells reached ∼70% confluent for transfection. Three plasmids at 10:10:1 ratio were mixed in Opti-MEM I Reduced Serum Medium (GIBCO) with TransIT-LT1 transfection reagent for 30 min at room temperature. The transfection mix was drop-wise added to the HEK293T cells. On day 5, lentivirus was collected from the supernatant and filtered through 0.22 μm filters. The lentivirus was directly added to cells for transduction or stored at –80°C.

### TEAR-CoV transfection of HEK293T cells

The gRNAs targeting SARS-CoV-2 or IAV for StCsm complex were synthesized by Integrated DNA Technologies. The StCsm proteins were purified as above-described. One day prior to transfection, HEK293T cells were plated at a density of 40 000 cells/well in 12-well plates. On the day of transfection, the StCsm proteins were mixed with gRNA for 15 min to assembled TEAR-CoV. The HEK293T cells were transfected using LipofectAmine™ CRISPRMAX™ Transfection Reagent (Thermo Fisher Scientific) according to the instruction with 0–6.0 μg of StCsm complex and 2 μg gRNA. After overnight transfection, the cells were used for challenge experiments.

### SARS-CoV-2 CTD and SARS-CoV-2-RdRP-GFP lentivirus challenge

After overnight incubation for TEAR-CoV transfection of HEK293T cells, the wild-type or TEAR-CoV-transfected HEK293T cells in the 12-well plates were switched to fresh medium and transfected with SARS-CoV-2-CTD plasmids or were challenged with SARS-CoV2-RdRP-GFP lentivirus. Twenty-four hours later, cells were tested by quantitative real-time PCR (qRT-PCR) on a Bio-Rad CFX Connect™ Real-Time System, flow cytometry on a BD FAC-Symphony flow cytometer (BD Biosciences), and western blotting.

### IAV challenge experiments

On day 1, HEK293T cells were plated onto 12-well plates at a density of 40 000 cells/well. Next day, TEAR-CoV transfection of HEK293T cells was performed using LipofectAmine™ CRISPRMAX™ Transfection Reagent (Thermo Fisher Scientific) according to the manufacturer's instructions. After overnight transfection, the wild-type or TEAR-CoV-transfected HEK293T cells in the 12-well plates were challenged overnight with IAV at MOI = 0.01 and 0.5. To determine the quantity of IAV that replicated and subsequently secreted from cells, we measured IAV RNA levels in the supernatant of infected cell cultures. The supernatants were heated at 95°C for 10 min to inactivate nucleases and viral particles. The heat inactivated cell supernatants were used to perform qRT-PCR. A standard curve of 1:10 dilutions of IAV from 10^7^ to 10 copies/μl was generated to quantify the titers of IAV in the cell supernatants. Normalized viral RNA levels for TEAR-CoV treatment were calculated as each sample's viral RNA quantity in copies, divided by the median viral RNA quantity determined by qRT-PCR of IAV without biotherapy (controls).

### IAV infection in mice

C57BL/6N mice (6–8 weeks) were purchased from ENVIGO (Indianapolis, IN). The mice were maintained in the animal facility at the University of North Dakota for at least two weeks before the experiment. Both males and females of mice were used randomly. All animal studies were approved by the University of North Dakota Institutional Animal Care and Use Committee and performed following the animal care and institutional guidelines. For *in vivo* analyses, we designed and constructed of Csm complex and gRNA expression vectors. To generate a Csm complex expression vector, coding sequence of Csm1–2–3–4–5 was codon optimized and the whole sequence was synthesized and inserted into pcDNA3.1(+) vector, *ori* (origin of replication: ColE1, f1, SV40), neomycin/kanamycin resistance, using BamH I and Xba I by Gene Universal service (Newark, DE), forming the pcDNA-CMV-CSM1–2–3–4–5 plasmid. To create gRNA expression vector, U6 promoter sequence, gRNA sequence and terminator T was also synthesized and inserted into pcDNA3.1/Zeo(+) with the bleomycin/ampicillin resistance through BamH I and Not I by Gene Universal service, forming pcDNA/Zeo-U6-Type III-gRNA plasmid. Next, C57BL/6N mice were infected intranasally with 100× TCID_50_ IAV using 15 μl under anesthesia by i.m. injecting mixture of ketamine (80 mg/kg) and xylazine (10 mg/kg). Six hours after infection, the mice were treated with TEAR-CoV plasmids (pcDNA-CMV-CSM1–2–3–4–5 and pcDNA/Zeo-U6-Type III-gRNA) through TurboFect *in vivo* Transfection Reagent (Thermo Fisher Scientific) according to the manufacture's instruction by i.m. injection. Two days later, the mice were euthanized and the lungs were harvested into PBS for further analyses, including viral load quantification and histological assays.

### RNA isolation and quantitative real-time PCR

Total RNA was isolated by using the Direct-zol™ RNA MiniPrep kit, followed with DNase I treatment. 2 μg of total RNA were used to cDNA synthesis using the High Capacity cDNA Reverse Transcription Kit (Thermo Fisher Scientific). qRT-PCR primers were ordered from Eurofins Genomics. qPCR was performed using Maxima SYBR Green qPCR Master Mix (Thermo Fisher Scientific) and run on CFX Connect™ Real-Time System (Bio-Rad). For SARS-CoV-2 qPCR measurements, primers against the RdPR and S segment were used. To measure IAV RNA, primers against the nucleocapsid (N) that had been validated previously, were used (7). A melt curve was analyzed at the end of each experiment to confirm specific amplification. The relative RNA abundance was calculated with the normalization of internal control GAPDH (21,22). To quantify the number of copies of viral RNA, a standard curve of 1:10 dilutions of IAV PCR target fragments from 10^7^ to 10 copies per microliter was used. Normalized viral RNA levels for TEAR-CoV treatment were calculated as each sample's viral RNA quantity determined by qPCR compared with IAV alone (controls).

### Western blotting analysis

The samples were separated by 10% SDS-PAGE and transferred to nitrocellulose members (GE). Membranes were incubated with mouse monoclonal antibody against GFP (Biolegend, San Diego, CA) and GAPDH at 1:5000 for overnight at 4°C. After three times washing with washing buffer, we added the corresponding secondary antibodies and incubated for 1.5 h at room temperature. Following five times washing, the protein bands were visualized by chemiluminescence (23,24).

### Flow cytometry

Cells were washed three times with PBS, treated with trypsin-EDTA, and fixed in 4% paraformaldehyde for 10 min. Cells were washed with FACS buffer (1× PBS, 0.3% BSA, 1 mM EDTA). FAC-Symphony flow cytometer (BD Biosciences) was used to collect raw data and data was analyzed using FlowJo software.

### Histological analysis

Lung tissues were fixed in 10% formalin (Sigma-Aldrich, St Louis, MO) for 48 h at 4°C and then embedded in paraffin using a routine histologic procedure. H&E staining was carried out according to standard staining procedure.

### Statistical analysis

We described in the Figure Legends for description of sample sizes and statistical analyses. One-way ANOVA plus Tukey's post hoc tests were used for comparisons using GraphPad software (GraphPad Software, La Jolla, CA). *P* > 0.05 was considered no significant difference, and *P* < 0.05 were considered statistically significant.

## RESULTS

### Design of TEAR-CoV and targetable regions for degradation of SARS-CoV-2 sequence *in vitro*

To develop a novel, timely therapeutic strategy to treat COVID-19 patients, we designed Type III CRISPR endonuclease antivirals for coronaviruses (TEAR-CoV), derived from *Streptococcus thermophilus* Type III CRISPR-Cas (11), to control SARS-CoV-2 infection (Figure 1A). TEAR-CoV system employs StCsm complex with Cas10–Csm2–Cms3–Csm4–Csm5 with a guide RNA (gRNA) to trigger targeting SARS-CoV-2 RNA genome and transcript degradation (Figure 1A). By removing activated ancillary nuclease Csm6, TEAR-CoV should not trigger non-specific RNA cleavage activity of Csm6 (18,25), and our data showed that TEAR-CoV did not cause degradation of bystander transcripts and cellular toxicity to the host (Supplementary Figure S1).

To evaluate the potential for a programmable antiviral platform for targeting ssRNA viruses, we sought to determine the effects of TEAR-CoV on cleaving SARS-CoV-2 sequences and potentially inhibiting SARS-CoV-2 growth and infection. We used a computational pipeline to search gRNA candidates targeting the conserved regions in the genomes of SARS-CoV-2 strains derived from Asia, South America, North America, Europe, Oceania, and Africa along with SARS-CoV and MERS-CoV (Supplementary Figure S2) (6,7). We found highly conserved regions between SARS-CoV-2, as well as SARS-CoV and MERS-CoV genomes (Figure 1B). We generated a collection of possible gRNAs targeting the highly conserved SARS-CoV-2 region (Figure 1C, Supplementary Table S1). To account for possible off-target effects, we used all possible gRNAs to predict off-target sites in human transcriptome and removed the off-targeting sequences ($ \le 5{\rm{\ }}$ mismatches). We obtained a collection of 1396 gRNAs targeting conserved regions in all SARS-CoV-2 (Figure 1B and C). To devise a strategy to target multiple viral components and sufficiently block SARS-CoV-2 infection, we have performed a computing analysis and discovered critical guide RNAs that target highly conserved sequences of Spike (S) protein and functional RdRP (Figure 1B), which is sufficient to block SARS-CoV-2 viral entry and replication (Figure 1A). Thereafter, we used TEAR-CoV, in which StCsm complexes (Supplementary Figure S3) were loaded with gRNA1 or gRNA2 targeting RdRP and gRNA3 or gRNA4 targeting S coding regions (Supplementary Table S2), to target and cleave the SARS-CoV-2 RNA genome. As expected, TEAR-CoV effectively cleaved the SARS-CoV-2 RNA genome (Figure 1D).

Next, we used Cell-Free Protein Expression platform—myTXTL to investigate TEAR-CoV’s potency in inhibiting SARS-CoV-2 protein expression (26). The plasmids encoding Csm complex and gRNA-targeted S mRNAs were added to myTXTL mix along with S fragment-deGFP (S fragment fused to GFP) reporter to determine GFP protein translation levels as described in the methods section (Figure 1E). Remarkably, we observed that TEAR-CoV repressed GFP expression by 62.9% for gRNA3 and 71.5% for gRNA4, respectively, compared to non-targeting gRNA during observation period of 15 h (Figure 1E).

Taken together, these findings indicate that TEAR-CoV may serve as an approach to disrupt SARS-CoV-2 genome and inhibit gene/protein expression.

### TEAR-CoV enables RNA knockdown in cell culture

**Figure 2.**
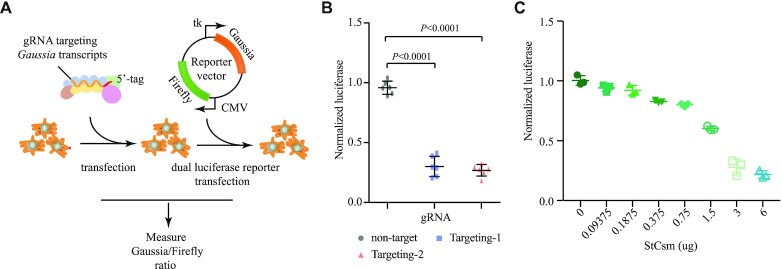
TEAR-CoV permits RNA-Guided RNA cleavage in human cells. (**A**) Procedure of RNA cleavage experiments in HEK293T cells. TEAR-CoV activity by gRNA against *Gaussia*. (**B**) Quantification of RNA cleavage of *Gaussia* luciferase using the TEAR-CoV in human cells HEK193T. Dots represent six biological replicates, error bars indicate SEM, one-way ANOVA plus Tukey post hoc test and *P* < 0.05 was considered statistically significant. (**C**) Optimization of TEAR-CoV of RNA targeting efficiency. Increasing amounts of StCsm complex were used to evaluate the ratio of *Gaussia* luciferase knockdown by TEAR-CoV (as measured by luciferase activity).

Direct delivery of proteins of TEAR-CoV to mammalian cells offers an alternative approach to plasmid expression with fast kinetics, which could be advantageous for specific therapeutic design. To assess RNA targeting activity of TEAR-CoV in human cells, we introduced a dual-luciferase reporter into the human embryonic kidney HEK293T cell line. StCsm/gRNA complex, gRNA targeting transcripts of Gaussia reporter, were transfected with LipofectAmine™ CRISPRMAX™ Transfection Reagents, which showed that TEAR-CoV reduced transcript levels of *Gaussia* by 65.8% and 69.0% (Figure 2A and B), respectively.

Next, we attempted to optimize TEAR-CoV by varying the amount of StCsm complex. The efficiency of *Gaussia* disruption positively correlated with StCsm complex abundance, increasing from 5.8% to 78.2% when the StCsm complex was increased from 0.09 to 6 μg (Figure 2C). These findings demonstrate that TEAR-CoV-based RNA engineering approach is RNA-guided specific transcript knockdown in eukaryotic cells, which is consistent with previous research that Type III CRISPR systems from both archaea and the same well-characterized bacterium (*S. thermophilus*) can mediate RNA transcript knockdown *in vivo* with *Sulfolobales* (27,28) or *zebrafish* (29), respectively. These recent developments may indicate the potential application of Type III CRISPR for mammalian or human gene editing in the future.

### TEAR-CoV is capable of inhibiting SARS-CoV-2 gene expression in human cells

**Figure 3.**
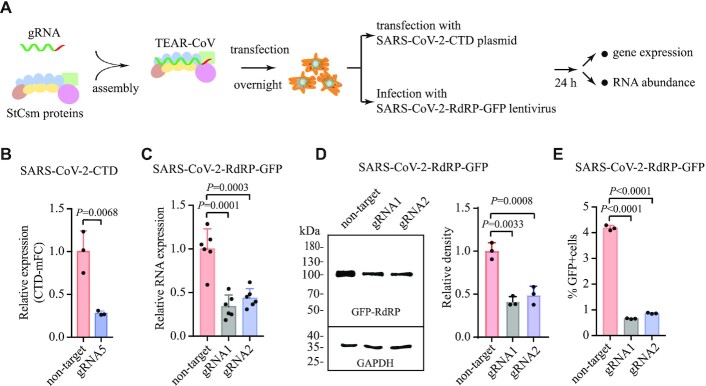
TEAR-CoV inhibits SARS-CoV-2 gene expression in mammalian cells. (**A**) Schematic illustrating TEAR-CoV targeting and cleavage of C-terminal domain (CTD) of SARS-CoV-2 S mRNA and RdRP of SARS-CoV-2-RdRP-GFP lentivirus. (**B**) qRT-PCR detecting expression levels of the CTD of S1 subunit (called RBD domain) of SARS-CoV-2 S when TEAR-CoV with gRNA5 targeting CTD sequence was delivered. Relative RNA expression was calculated by normalizing to GAPDH. Bars indicate mean SEM, one-way ANOVA plus Tukey post hoc test and *P* < 0.05 was considered statistically significant. (**C)** TEAR-CoV affecting mRNA abundance as measured by qRT-PCR when SARS-CoV-2-RdRP-GFP was delivered via lentiviral transduction. One-way ANOVA plus Tukey post hoc test and *P* < 0.05 was considered statistically significant. (**D**, **E**) GFP expression as measured by western blot (D) and flow cytometry (E) when SARS-CoV-2-RdRP-GFP was delivered via lentiviral transduction. One-way ANOVA plus Tukey post hoc test and *P* < 0.05 was considered statistically significant.

Next, we sought to evaluate whether TEAR-CoV may be used for targeting and cleaving SARS-CoV-2 genes in human cells. As a recent study indicates that the CTD (C-terminal domain of S1 subunit, also called receptor binding domain [RBD]) of S protein of SARS-CoV2, is critical for binding to its receptor ACE2 in human epithelial cells (30). RBD targeting is widely recognized a safer and effective vaccine approach. To this end, we first transfected assembled TEAR-CoV with gRNA5 targeting CTD mRNA sequence and SARS-CoV2-CTD plasmid expressing CTD domain (30) into HEK293T cells (Figure 3A). We observed that TEAR-CoV repressed SARS-CoV-2 CTD, causing a 72% inhibition of transcription (Figure 3B).

We further validated the effect of TEAR-CoV with gRNA1 or gRNA2-mediated RdRP repression when SARS-CoV-2-RdRP-GFP expressing a synthesized fragment (peptide) of SARS-CoV-2 RdRP fused to GFP was introduced via lentiviral transduction (6), which would reflect some characteristics of natural SARS-CoV-2 infection, such as causing inflammatory responses (a chief feature of COVID-19 long-haulers) (Figure 3A). We observed that the RdRP fragment expression was repressed by 66% for gRNA1 and 56.3% for gRNA2, respectively (Figure 3C). We further validated TEAR-CoV-mediated repression of SARS-CoV-2-RdRP-GFP reporters by detecting the fused RdRP-GFP protein expression (Figure 3D), and observed approximately 6.4- and 4.8-fold decrease in GFP by flow cytometry analysis for gRNA1 and gRNA2, respectively (Figure 3E). These results, collectively, suggest that TEAR-CoV may be an alternative approach to target and degrade any chosen SARS-CoV-2 sequence in human cells.

### TEAR-CoV possesses antiviral activity against live IAV in human cells and in mice

**Figure 4.**
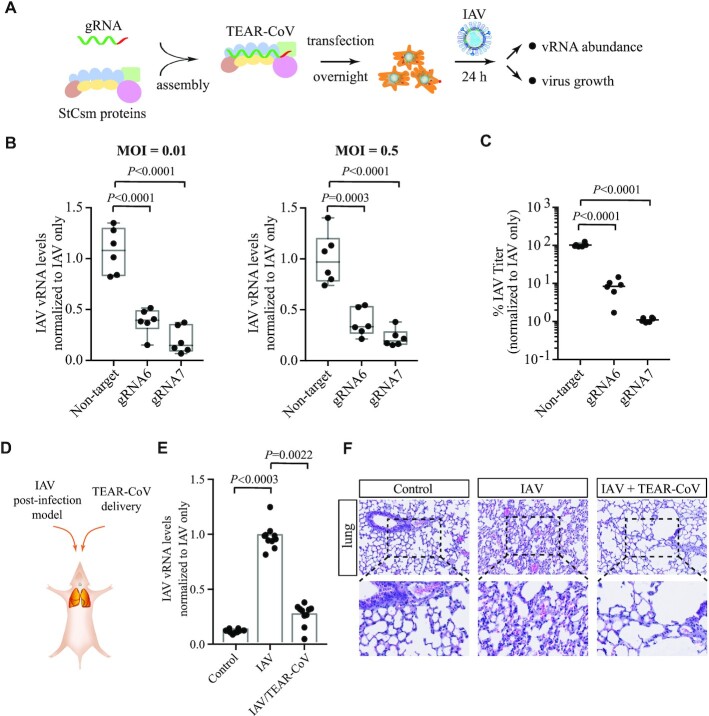
TEAR-CoV possesses antiviral activity against live IAV infection in human cells and in mice. (**A**) Schematic illustrating the use of TEAR-CoV for the defense of influenza A virus (IAV) infection. (**B**) Normalized IVA RNA levels when transfection of TEAR-CoV with pilot non-targeting control gRNA or gRNA6 and gRNA7 targeting the IVA mRNA and complementary viral RNA that is nucleocapsid protein (NP) gene in genomic segment 5. Boxplots: center line represents median, whiskers represent minimum to maximum, and dots represent six independent replicates; one-way ANOVA plus Tukey test. (**C**) Comparison of viral replication when TEAR-CoV were delivered at MOI = 0.01. One-way ANOVA plus Tukey post hoc test and *P* < 0.05 was considered statistically significant. (**D**) Schematic depiction of TEAR-CoV treatment for IAV post-infection mouse model. (**E**) Normalized IAV RNA levels after transfection of TEAR-CoV into IAV-infected mice. (**F**) H&E staining assessing lung injury in mice with IAV infection with or without TEAR-CoV treatment.

To validate the results and broaden the application for TEAR-CoV targeting live virus, we next applied our TEAR-CoV strategy in targeting therapy of influenza A virus (IAV), a negative sense ssRNA pandemic causing virus (Figure 4A) (31). We designed IAV-targeted gRNAs to target both mRNA and the complementary viral RNA that gRNA6 and gRNA7 target the nucleocapsid (N) gene in genomic segment 5; and nucleocapsid protein is required for viral packaging. We examined antiviral activity of TEAR-CoV against IAV (A/Puerto Rico/8/1934 (PR8)) infection at different MOIs (multiplicity of infection, 0.01 and 0.5) in human cells. TEAR-CoV-mediated targeting decreased IAV viral RNA levels by 69% for gRNA6 and 87% for gRNA7 (MOI = 0.01, Figure 4B). Importantly, we noticed similar reduction in IAV viral RNA, at a higher MOI (0.5), 62.9% for gRNA6 and 78% for gRNA7 (Figure 4B), indicating that TEAR-CoV may be more effective in inhibiting IAV infection at a higher viral load. Moreover, there was a > 3 log_2_ and > 6 log_2_ reduction in IAV viral titer for gRNA6 and gRNA7, respectively, compared to non-target gRNA (Figure 4C).

Next, we employed a mouse IAV infection model to explore the therapeutic effects of TEAR-CoV (Figure 4D). After IAV infection, TEAR-CoV was delivered through intranasal instillation, we analyzed IAV viral titer, which showed that TEAR-CoV significantly reduced the survival rates of IAV virus *in vivo* (Figure 4E). Furthermore, TEAR-CoV significantly reduced tissue damage and inflammatory responses in mice by immunohistochemistry staining (Figure 4F), illustrating that TEAR-CoV may serve as an alternative therapeutic strategy with safe and strong efficacy shown in animals.

Taken together, these data reveal that TEAR-CoV efficiently cleaves viral RNA in live IAV infection, providing a proof-of-concept that TEAR-CoV may be applicable in controlling live SARS-CoV-2 infection in clinical settings.

### TEAR-CoV as a potential antiviral platform against human coronaviruses

**Figure 5.**
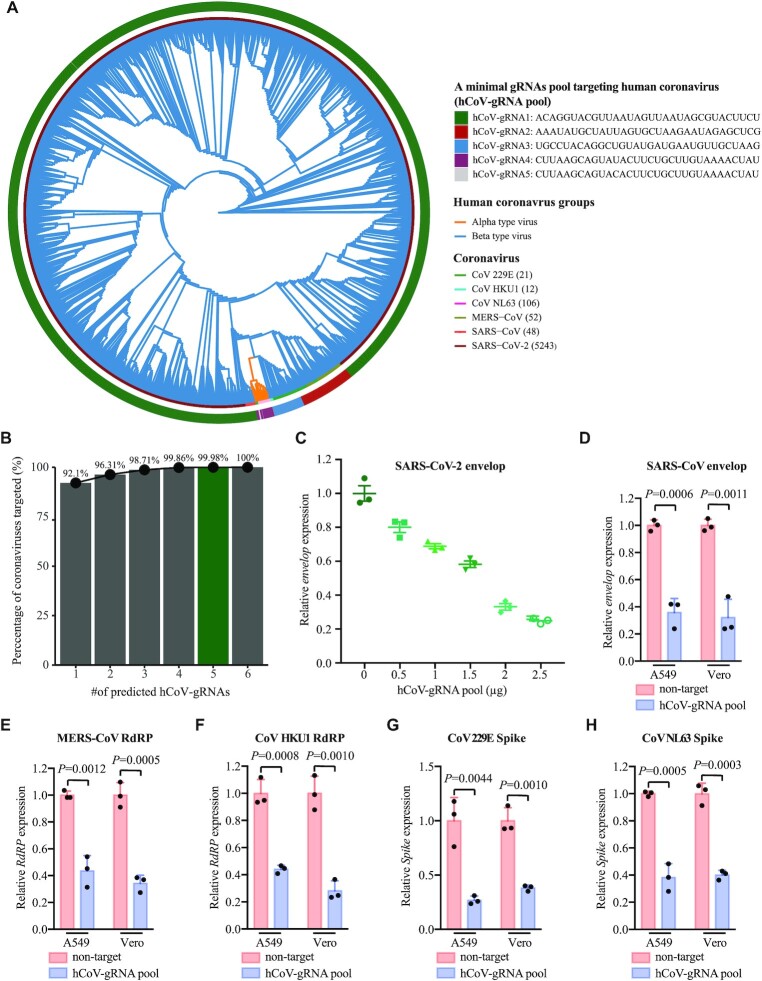
TEAR-CoV enables rapid design of prophylactics and therapeutics targeting any coronaviruses. (**A**) Phylogenetic tree of human coronaviruses constructed by the complete reference sequences. The outer ring shows coverage by each of gRNAs (hCoV-gRNA1 to hCoV-gRNA5) targeting almost all human coronaviruses’ genomes. The inner ring shows current species of human coronaviruses that infect humans, including SARS-CoV-2. (**B**) Prediction of minimal numbers of hCoV-gRNAs targeting the human coronaviruses reference genomes. (**C**) qRT-PCR detecting expression levels of the SARS-CoV-2 envelope when TEAR-CoV with hCoV-gRNA pool was delivered. Relative RNA expression was calculated by normalizing to GAPDH. Bars indicate mean SEM, one-way ANOVA plus Tukey post hoc test and *P* < 0.05 was considered statistically significant. (**D–H**) qRT-PCR detecting expression levels of human coronaviruses by TEAR-CoV with hCoV-gRNA pools. Human coronaviruses: SARS-CoV (D), MERS-CoV (E), CoV KHU1 (F), CoV 229E (G) and CoV NL63 (H). Relative RNA expression was calculated by normalizing to GAPDH. Bars indicate mean SEM, one-way ANOVA plus Tukey post hoc test and *P* < 0.05 was considered statistically significant.

As humans have witnessed three catastrophic pandemics caused by the human coronaviruses within two decades, we have made computing prediction to identify minimal targeting sites for TEAR-CoV to inhibit almost all human coronaviruses, which may serve as strategic preparedness for next pandemics caused by the same family of viruses. To do this, we analyzed 5506 known human coronaviruses’ genomes (Figure 5A), including 5243 SARS-CoV-2, to screen the gRNAs for TEAR-CoV targeting a majority of coronavirus genomes (Supplementary Figure S4). With perfect matches between gRNAs and a part of the human coronavirus genome, one gRNA (hCoV-gRNA1) could target the conserved regions of 92.1% of coronavirus genomes (Figure 5A and B, Supplementary Table S3), which encoded the envelope protein required for SARS-CoV-2 packaging; this is due to the major target sites by hCoV-gRNA1 is on the SARS-CoV-2 reference genomes. We found that a combination of only five hCoV-gRNAs could target almost all (99.8%) of human coronavirus genomes and six hCoV-gRNAs covered all human coronavirus reference genomes as reported thus far (data generated on NCBI database on 26 July 2020, and ViPR database on 30 July 2020) (Figure 5A and B, Supplementary Table S3). Given the extreme diversity and rapid evolution of coronaviruses, using a small number of gRNA pools driven by TEAR-CoV to target a wide range of coronavirus species and strains further highlights the unique power of our method vs. the traditional therapeutic approaches.

We generated a map that hCoV-gRNA pool targets the currently characterized human coronaviruses (Figure 5A), including MERS-CoV, SARS-CoV, CoV 229E, CoV HKU1, CoV NL63 and SARS-CoV-2. To investigate whether hCoV-gRNA pools with TEAR-CoV against SARS-CoV-2, we validated the effect of TEAR-CoV with hCoV-gRNA pool-mediated inhibition of expression of SARS-CoV-2 envelope (Figure 5C), suggesting that TEAR-CoV together with this gRNA pool can potentially be applied to target a wide range of SARS-CoV-2 strains. To this end, we tested TEAR-CoV’s antiviral effects against distinct diverse sequences of different coronaviruses, CoV 229E, HKU1, NL63, MERS-CoV or SARS-CoV. hCoV-gRNA pool targeting the envelope of SARS-CoV resulted in approximate 64.2% and 67.9% reduction in envelope transcripts in A549 and Vero cells (Figure 5D), respectively. hCoV-gRNA pool targeting RdRP of MERS-CoV and CoV HKU1 led to inhibition in RdRP transcription (MERS-CoV: 56.5% in A549 cells and 65.8% in Vero cells; CoV HKU1: 56.0% in A549 cells and 71.8% in Vero cells) (Figure 5E and F). hCoV-gRAN pool targeting of CoV 229E and NL63 Spike protein could also repress its expression (CoV 229E: 73.1% in A549 cells and 61.9% in Vero cells; CoV NL63: 61.7% in A549 cells and 59.9% in Vero cells) (Figure 5G and H). Taken together, TEAR-CoV with a hCoV-gRNA pool can be an effective system to target and degrade SARS-CoV-2 and a broad range of human coronavirus in human cells.

## DISCUSSION

We have designed and constructed a CRISPR-based therapeutic as a potential approach for combating SARS-CoV-2 and IAV. Our work showed both the transcripts and genome of SARS-CoV-2 RNA can be specifically targeted and degraded by TEAR-CoV. In addition, replication of certain virulence transcripts is also inhibited by TEAR-CoV. Further, TEAR-CoV may effectively reduce live IAV infection in human cells and in mice. Moreover, our bioinformatics data reveal that as few as a combination of five hCoV-gRNAs are able to target 99.98% of human coronavirus and validated representative targeting and cleavage of common human coronaviruses by TEAR-CoV. It is remarkable that the TEAR-CoV strategy may have the potential for therapeutic application in a wide variety of human ssRNA pathogens. As the recent frequent occurrence of devastating pandemics is an ultimate concern for the CRISPR biotechnology in infectious diseases, our combination approach may offer the improved option for controlling emerging viral infection, where the suffering patients are in dire need of therapy.

In the native environment, Type III CRISPR-Cas interference typically eradicates not only the specific targeted foreign RNA but also subsequent non-specific RNA (11,12,17,25). In this study, TEAR-CoV derived from Type III CRISPR-Cas systems triggers sequence-specific degradation of a target RNA, avoiding possible degradation of bystander transcripts and cellular toxicity, which allows us to observe the specific RNA knockdown outcome for Type III CRISPR in a heterologous eukaryotic context. Thus, applications for targeting mammalian RNA with Type III CRISPR will be realized at an enhanced scale in the coming years for programmable RNA targeting. Moreover, the targeted inhibition of RdRP and Spike proteins, essential for coronavirus replication and/or receptor binding (32–37), of SARS-CoV-2 by TEAR-CoV is responsible for disabling virus production and infectivity. It is important to note that TEAR-CoV may also be used to reduce live IAV infectivity, a viral pathogen with high seasonal prevalence potential, proving a proof-of-concept antiviral strategy for robustly and broadly against SARS-CoV-2 by TEAR-CoV. At present, development of FDA-approved vaccines or antivirals remains challenging for a new type of coronavirus pandemics, the approach of TEAR-CoV offers a promising new solution to this unmet need. Thus, next step will investigate the efficiency and specificity of gRNAs for inhibition infection or respiratory tract cells with live SARS-CoV-2 virus using a biosafety level 3 facility.

The nuclease activities of Type III CRISPR-Cas system through RNA targeting mechanisms are not restricted to intruder RNAs, but also can induce cell death through nonspecific degradation of cellular ssDNA (as a suicide-switch) (12,13,18). Internal flexibility for off-switch activity of nonspecific ssDNA cleavage by Cas10′s enzymatic reactions may be exquisitely controlled by designing anti-tag RNA substrate (13,15,38). However, this will strongly decrease the number of available targets in SARS-CoV-2 or other RNA viruses. Prior studies have shown that the activation of HD nuclease in the Cas10 subunit plays a critical role for allosteric activation of DNase activity (38). Mutations of Q266, R397, H412, Y424, K495 and K617 in the HD nuclease domain greatly impaired Cas10 DNase activity (38), supporting that knocking-out the HD nuclease activity by mutation of key residues may inhibit ssDNA degradation. Therefore, it is intriguing to further investigate mutations at selected residues of Cas10 subunit in the TEAR-CoV, which should help reduce potential non-specificities of TEAR-CoV and avoid degrading the human genome and autoimmunity.

Any approach that targets RNA sequences with nucleotide sensitivity will potentially subject to viral evasion through mutations. For Type I and II CRISPR-Cas systems, carrying a single-nucleotide mutation in the protospacer adjacent motif (PAM) or seed causes immune failure and results in viral escape (39,40). Type III-A CRISPR-Cas targeting tolerates sequence changes with protospacer or upstream of the protospacer (15), and single-nucleotide substitutions do not impair Type III-A CRISPR-Cas immunity (15,41). Nevertheless, accumulation of mismatches in the first five nucleotides of the protospacer abrogates Type III-A CRISPR-Cas immunity (15). In agreement with this, the most frequent event that can lead to escape from Type III-A CRISPR-Cas immunity is the deletion of the target sequence from the viral genome. Type III-A CRISPR-Cas immunity is impossible to escape if the target is located in an indispensable gene that cannot tolerate deletions, which could result in viral extinction. For TEAR-CoV, based on Type III-A CRISPR-based RNA editing by using computational design, we have established a robust set of gRNAs targeting the highly conserved regions, especially essential *RdRP* or *Spike* genes, minimizing loss of inhibition activity of TEAR-CoV via viral mutational escape. Moreover, to prevent the takeover of viral escape mutants, we may use multiple guide RNAs (42), which can be readily designed by adjusting the CRISPR arrays. Previous studies showed that targeting RNA structures has an effect on nucleic acid-based antiviral approaches (43). This indicates that human coronaviruses may be less susceptible to inhibition by TEAR-CoV due to the secondary structure of RNA sequence or coating with viral/host protective protein. Although our results reveal that cleavage of live IAV and SARS-CoV-2 genome is dependent on gRNAs by TEAR-CoV, the relationship between virus RNA structure and the activity of TEAR-CoV remain unknown. To overcome this difficulty, *in vivo* high-throughput screen of gRNAs may help identify highly effective hCoV-gRNAs targeting live SARS-CoV-2 or human coronaviruses. In addition, resolving the target RNA structures would help to evolve the target sites of viral genomic regions by TEAR-CoV. With optimization and further testing, TEAR-CoV may greatly accelerate the development of countermeasures for emerging viruses of pandemic potential.

We have demonstrated that TEAR-CoV is able to inhibit viral sequences *in vitro*, but therapeutic use in clinics will need to develop an effective and safe *in vivo* delivery method into human cells. TEAR-CoV may be an antiviral option for controlling SARS-CoV-2 to benefit the suffering human populations, when the CRISPR-based therapeutics may be directly delivered into human respiratory tract cells through a viral vector (i.e. AAV) (Supplementary Figure S5A) (44,45). Using hCoV-gRNAs as the guide, the TEAR-CoV may induce virus clearance to serve as prophylactic or treatment measures. If using the tissue-specific promoter or cell-type promoter driving gene expression, such as surfactant protein promoter (SP-C) (46), this will allow TEAR-CoV to protect the functional important type II pneumocytes (AT2) that are primary targets for SARS-CoV-2 infection (47). Moreover, the approach with TEAR-CoV consisting of synthesized hCoV-gRNAs and StCsm complex proteins may serve as a vehicle for specific delivery into certain human cells by nanoparticles or other methods that provide targeting specificity (Supplementary Figure S5B) (44,48). It is important to note that combined with the peptide-based technology could lead to Cas9–gRNA complexes rapidly entering into airway epithelia (49), providing the potential therapeutic avenues for delivering TEAR-CoV to human respiratory tract cells. Furthermore, TEAR-CoV and its cognate gRNAs could be delivered as lipid nanoparticles (LNP) that encapsulate modified mRNA (Supplementary Figure S5C) (50,51), which induce high levels of protein expression *in vivo*. Electroporation (EP) delivery of DNA molecules is an attractive approach (52). Although *in vivo* delivery for therapies remain many challenges (44), we anticipate that further testing could determine the suitable delivery for TEAR-CoV to control infections and enhance human health.

Our study represents the first step in the use of Type III CRISPR-based TEAR-CoV against viral replication and function. In the future, the safety and efficacy of TEAR-CoV warrant further study to improve this system and combat SARS-CoV-2 and other emerging viruses in animal models and humans. If successful, such advances would facilitate the development of Type III CRISPR-Cas systems in antiviral treatment.

## DATA AVAILABILITY

All data are available in the main text or the Supplementary Data.

## Supplementary Material

gkac016_Supplemental_Files
