# Supplementary material for: Type III CRISPR-based RNA editing for programmable control of SARS-CoV-2 and human coronaviruses
Source: Nucleic Acids Res. 2022 Feb 15;50(8):e47. doi: 10.1093/nar/gkac016 (PMC9071467; doi:10.1093/nar/gkac016)
Supplement: gkac016_Supplemental_Files [file gkac016_supplemental_files.zip › Supplemental Dates.pdf]

Supplementary data for

Type III CRISPR-based RNA editing for programmable control of SARS-CoV-2 and human coronaviruses

Ping Lin, Guanwang Shen, Kai Guo, Shugang Qin, Qinqin Pu, Zhihan Wang, Pan Gao, Zhenwei Xia, Nadeem Khan, Jianxin Jiang, Qingyou Xia, Min Wu

Supplemental figures

	character	degradation
**TEAR-CoV**	• specific-targeted RNA	➤ SARS-CoV-2 RNA genome cleavage
**type III system**	• specific-targeted RNA	➤ SARS-CoV-2 RNA genome cleavage
	• Non-specific RNA and DNA	➤ Human Cell transcripts degradation
**type VI system (Cas13d)**	• specific-targeted RNA	➤ SARS-CoV-2 RNA genome cleavage
	• Non-specific RNA	➤ Human Cell transcripts degradation

Supplementary Figure S1. Advantage and disadvantage of TEAR-CoV, type III, and type VI CRISPR-Cas systems

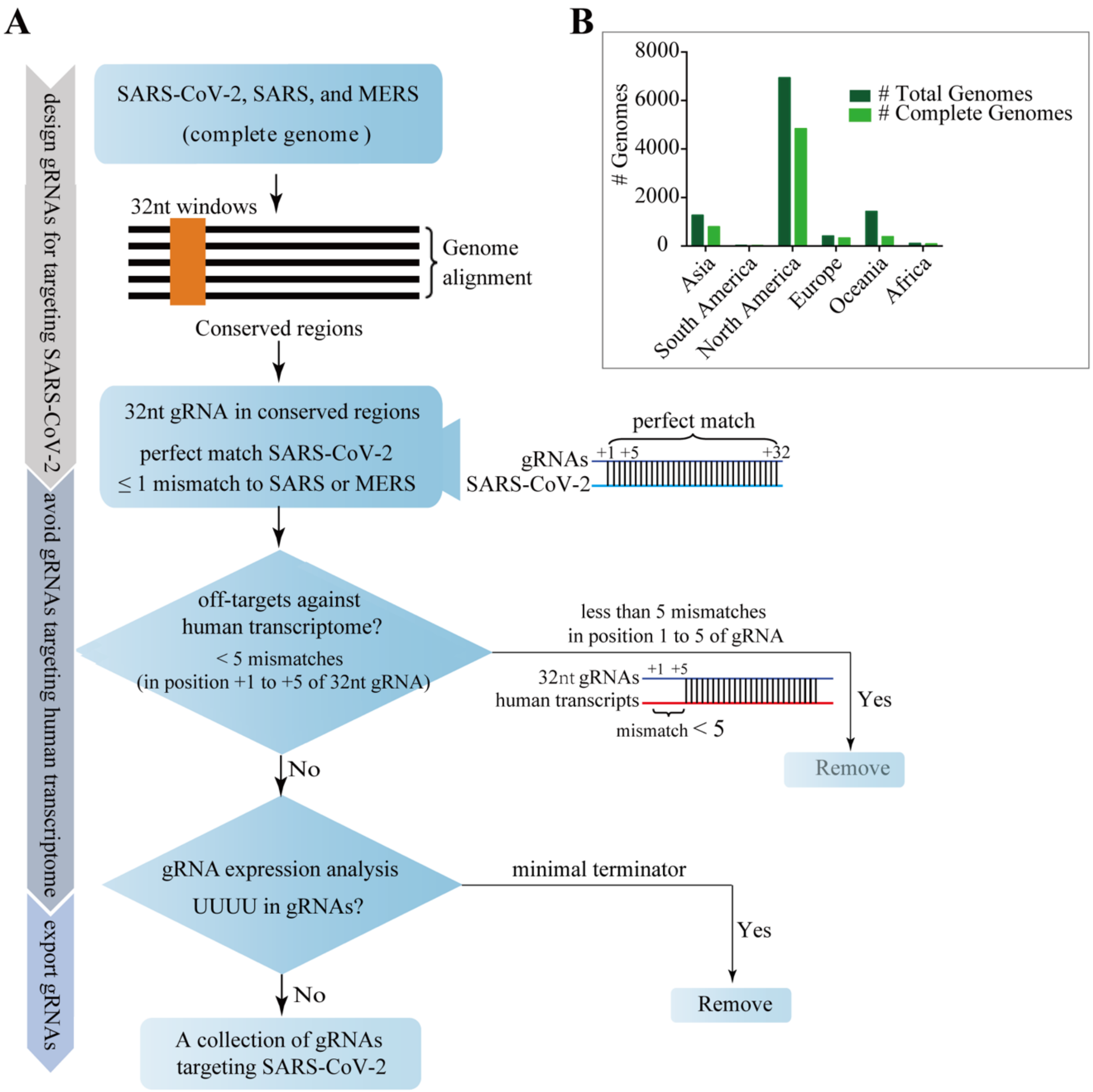

Supplementary Figure S2. Bioinformatic pipeline to predict all possible gRNA for SARS-CoV-2.
(**A**) Our workflow to analyze all predicted gRNAs targeting conserved regions between SARS-CoV-2, SARS, and MERS-CoV. (**B**) The number of complete SARS-CoV-2 genome sequences from Asia, South America (SA), North America (NA), Oceania, Europe, and Africa used for bioinformatic analysis.

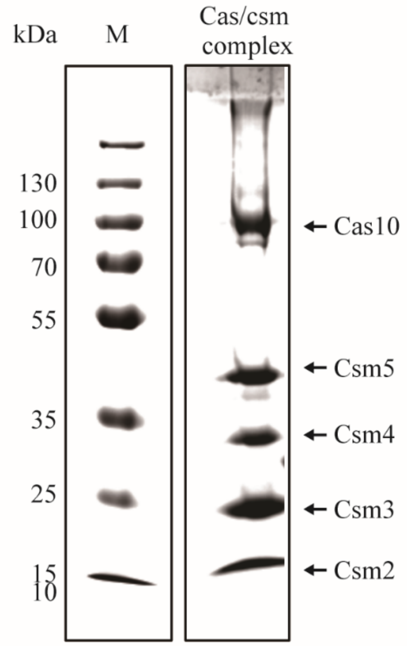

Protein	kDa
Cas10	87
Csm5	41
Csm4	34
Csm3	25
Csm2	14

Supplementary Figure S3. Purification of StCsm complex protein for TEAR-CoV.

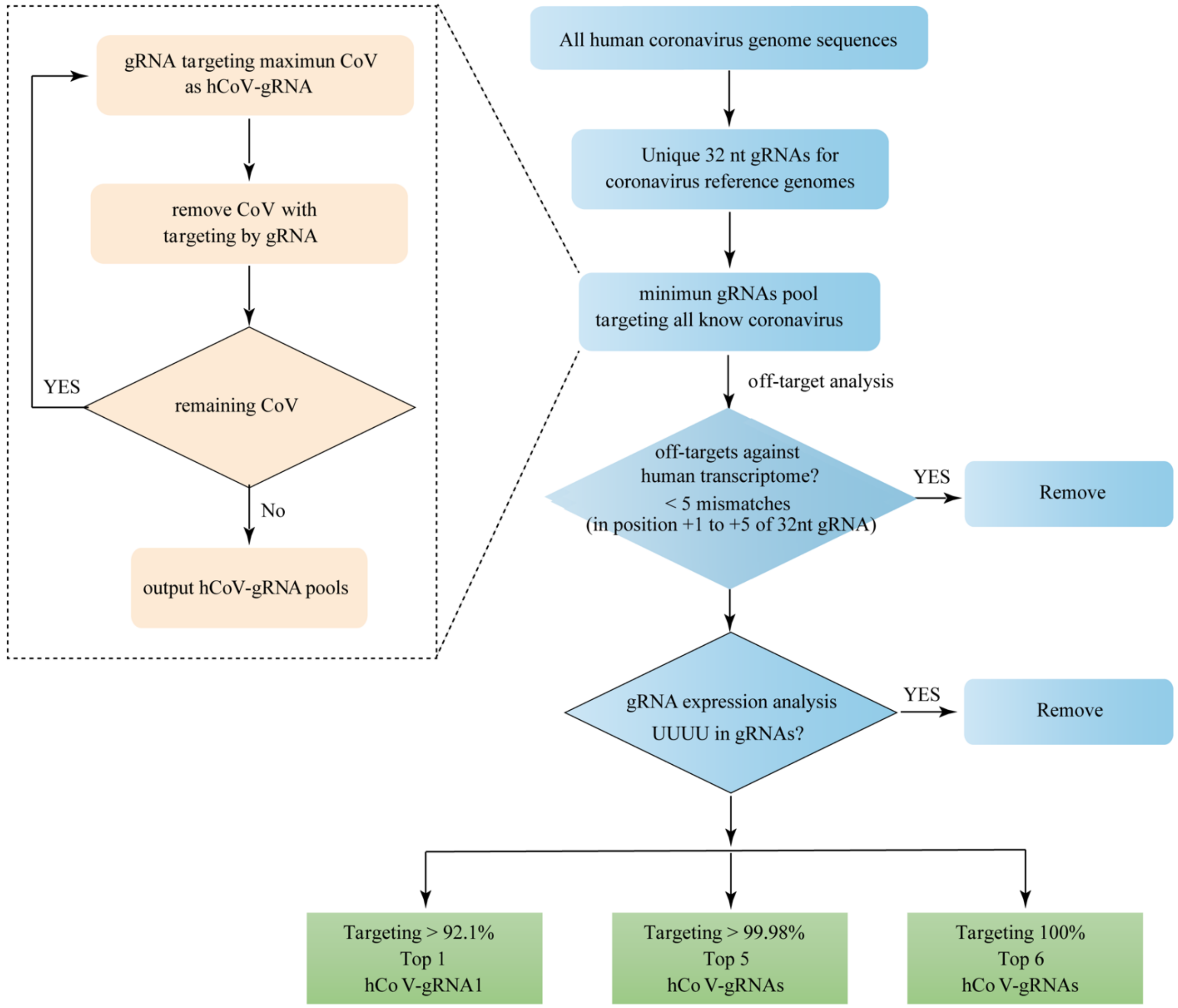

Supplementary Figure S4. Bioinformatic pipeline to predict a minimal gRNAs targeting all know human coronavirus.

Our workflow was used to analyze hCoV-gRNA pool targeting as many as human coronavirus. The number of hCoV-gRNAs was denoted in parentheses.

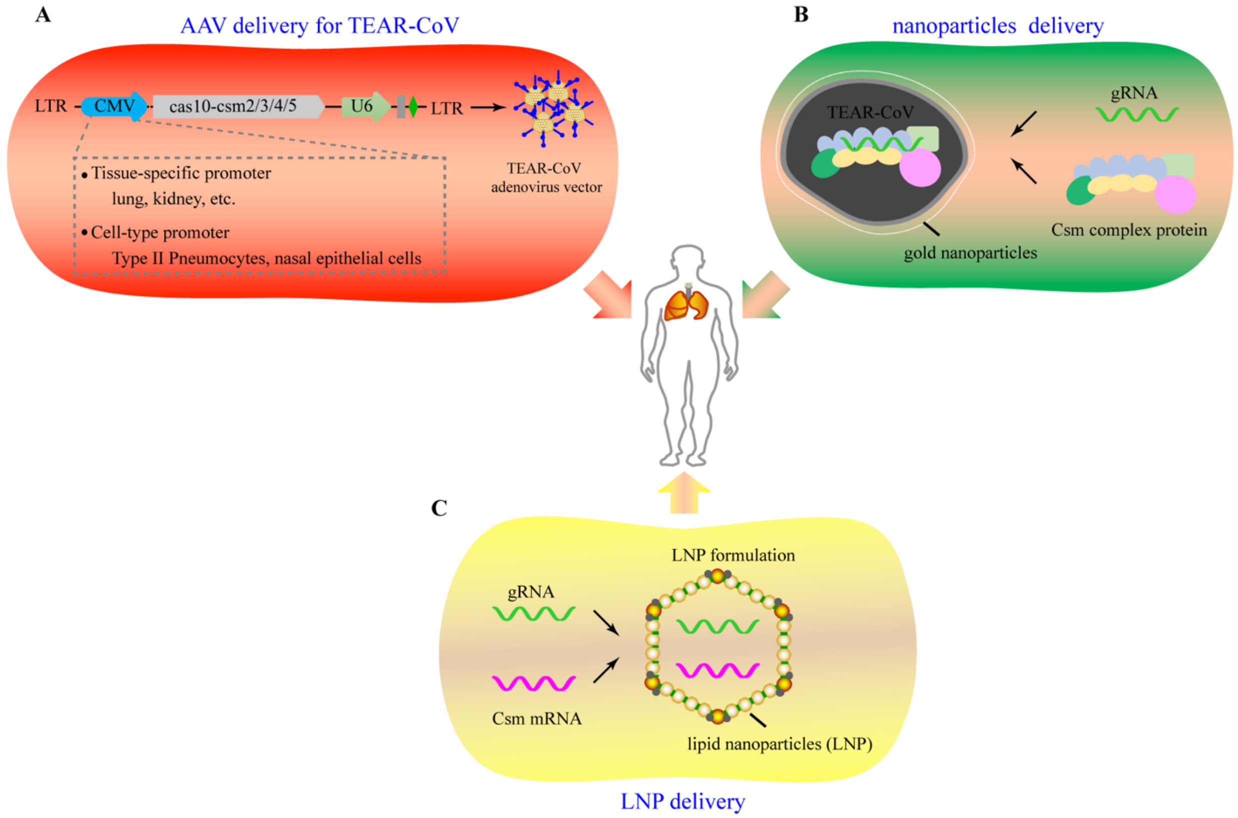

Supplementary Figure S5. Strategies for in vivo TEAR-CoV delivery.

(**A**) Schematic for AAV design carrying TEAR-CoV. (**B**) The approach of nanoparticles delivering TEAR-CoV consisting of synthesized hCoV-gRNAs and StCsm complex proteins into certain human cells for therapy of infection. (**C**) The modified mRNA of TEAR-CoV and its cognate gRNAs could be delivered as lipid nanoparticles (LNP).

Supplementary tables

Supplementary Table S1. Sequence of the potential gRNAs targeting the conserved regions of SARS-CoV-2 and their coordinates.

Supplementary Table S2. All sequences used in the study

**gRNA sequences targeting SARS-CoV-2**		
**Name**	**Spacer DNA sequence**	**target gene**
gRNA1	ACGTGATATATGTGGTACCATGTCACCGTCTA	RdRP
gRNA2	TATGTCTGATCCCAATATTTAAAATAACGGTC	RdRP
gRNA3	GATGAACCTGTTTGCGCATCTGTTATGAAATA	Spike
gRNA4	CCTTTATCAGAACCAGCACCAAAATGTATAAC	Spike
gRNA5	AAATGATGCGGAATTATATAGGACAGAATAAT	Spike
Non-targeting gRNA	GGACACGCTGAACTTGTGGCCGTTTACGTCGC	N/A
**gRNA sequences targeting IAV**		
**Name**	**Spacer DNA sequence**	**segment**
gRNA6	GTAATGAAGGATCTTATTTCTTCGGAGACAAT	5
gRNA7	GGTCGGTTGCTCACAAGTCCTGCCTGCCTGCC	5
**gRNA sequences targeting RNA substrates**		
**Name**	**sequence**	**target gene**
gRNA8	GGACACGCUGAACUUGUGGCCGUUUACGUCGC	RNA substrates
RNA substrate	GGGCGGCAAAUUGAGGAGACGGCGACGUAAACGGCC ACAAGUUCAGCGUGUCC	N/A
**Sequences for qRT-PCR detection**		
**Name**	**sequence**	
SARS-COV-2 S-CDT RT-qPCR (forward)	ACTTGTGCCCTTTTGGTGAAG	
SARS-COV-2 S-CDT RT-qPCR (reverse)	TTTGCCCTGGAGCGATTTGT	
SARS-COV-2-RdRP RT-qPCR (forward)	AACGGGTTTGCGGTGTAAGT	
SARS-COV-2-RdRP RT-qPCR (reverse)	TTTAGCAAAACCAGCTACTTTATCATTGTAG	
GAPDH RT-qPCR (forward)	TGCACCACCAACTGCTTAGC	
GAPDH RT-qPCR (reverse)	GGCATGGACTGTGGTCATGAG	
IAV RT-qPCR (forward)	CAAGCAGCAGAGGCCATGGA	
IAV RT-qPCR (reverse)	GACCAGCACTGGAGCTAGGA	

Supplementary Table S3. Prediction of minimal gRNA pools for targeting human coronaviruses (5,506)

Top five hCoV-gRNA pool sequences targeting 99.98% of human coronaviruses		
**gRNA name**	**Spacer DNA sequence**	**Spacer RNA sequence**
hCoV-gRNA1	ACAGGTACGTTAATAGTTAATAGCGTACTTCT	ACAGGUACGUUAAUAGUUAAUAGCGUACUUCU
hCoV-gRNA2	AAATATGCTATTAGTGCTAAGAATAGAGCTCG	AAAUAUGCUAUUAGUGCUAAGAAUAGAGCUCG
hCoV-gRNA3	TGCCTACAGGCTGTATGATGAATGTTGCTAAG	UGCCUACAGGCUGUAUGAUGAAUGUUGCUAAG
hCoV-gRNA4	CTTAAGCAGTATACTTCTGCTTGTAAAACTAT	CUUAAGCAGUAUACUUCUGCUUGUAAAACUAU
hCoV-gRNA5	CTTAAGCAGTACACTTCTGCTTGTAAAACTAT	CUUAAGCAGUACACUUCUGCUUGUAAAACUAU
**Top six hCoV-gRNA pool sequences targeting 100% human coronaviruses**		
**gRNA name**	**Spacer DNA sequence**	**Spacer RNA sequence**
hCoV-gRNA1	ACAGGTACGTTAATAGTTAATAGCGTACTTCT	ACAGGUACGUUAAUAGUUAAUAGCGUACUUCU
hCoV-gRNA2	AAATATGCTATTAGTGCTAAGAATAGAGCTCG	AAAUAUGCUAUUAGUGCUAAGAAUAGAGCUCG
hCoV-gRNA3	TGCCTACAGGCTGTATGATGAATGTTGCTAAG	UGCCUACAGGCUGUAUGAUGAAUGUUGCUAAG
hCoV-gRNA4	CTTAAGCAGTATACTTCTGCTTGTAAAACTAT	CUUAAGCAGUAUACUUCUGCUUGUAAAACUAU
hCoV-gRNA5	CTTAAGCAGTACACTTCTGCTTGTAAAACTAT	CUUAAGCAGUACACUUCUGCUUGUAAAACUAU
hCoV-gRNA6	GTGTCACTCGTGACATAGCATCTACAGATACT	GUGUCACUCGUGACAUAGCAUCUACAGAUACU
**Number of gRNAs**	**% of Targeted CoV Genomes**	**gRNA list in the category**
1	92.10%	hCoV-gRNA1
2	96.30%	hCoV-gRNA1|hCoV-gRNA2
3	98.71%	hCoV-gRNA1|hCoV-gRNA2|hCoV-gRNA3
4	99.86%	hCoV-gRNA1|hCoV-gRNA2|hCoV-gRNA3|hCoV-gRNA4
5	99.98%	hCoV-gRNA1|hCoV-gRNA2|hCoV-gRNA3|hCoV-gRNA4|hCoV-gRNA5
6	100.00%	hCoV-gRNA1|hCoV-gRNA2|hCoV-gRNA3|hCoV-gRNA4|hCoV-gRNA5|hCoV-gRNA6
